# Cybersecurity Enterprises Policies: A Comparative Study

**DOI:** 10.3390/s22020538

**Published:** 2022-01-11

**Authors:** Alok Mishra, Yehia Ibrahim Alzoubi, Asif Qumer Gill, Memoona Javeria Anwar

**Affiliations:** 1 Informatics and Digitalization, Molde University College—Specialized University in Logistics, 6410 Molde, Norway; 2 Department of Software Engineering, Atilim University, Ankara 06830, Turkey; 3 Management Information Systems Department, College of Business, American University of the Middle East, Egaila 15453, Kuwait; yehia.alzoubi@aum.edu.kw; 4 School of Computer Science, The University of Technology Sydney, 15 Broadway, Ultimo, NSW 2007, Australia; Asif.Gill@uts.edu.au (A.Q.G.); memoona.j.anwar@student.uts.edu.au (M.J.A.)

**Keywords:** cybersecurity (CS), cybersecurity polices, cyberspace, enterprise(s), information and communication technology (ICT)

## Abstract

Cybersecurity is a critical issue that must be prioritized not just by enterprises of all kinds, but also by national security. To safeguard an organization’s cyberenvironments, information, and communication technologies, many enterprises are investing substantially in cybersecurity these days. One part of the cyberdefense mechanism is building an enterprises’ security policies library, for consistent implementation of security controls. Significant and common cybersecurity policies of various enterprises are compared and explored in this study to provide robust and comprehensive cybersecurity knowledge that can be used in various enterprises. Several significant common security policies were identified and discussed in this comprehensive study. This study identified 10 common cybersecurity policy aspects in five enterprises: healthcare, finance, education, aviation, and e-commerce. We aimed to build a strong infrastructure in each business, and investigate the security laws and policies that apply to all businesses in each sector. Furthermore, the findings of this study reveal that the importance of cybersecurity requirements differ across multiple organizations. The choice and applicability of cybersecurity policies are determined by the type of information under control and the security requirements of organizations in relation to these policies.

## 1. Introduction

Cyberspace is a digital and virtual environment in which individuals may connect at any time and from any location by utilizing the Internet, computer networks, or other comparable tools. Cell phones, mobile telephones, iPhones, offline or online computer equipment, and any information saved or exchanged over computer networks, such as databases and electronic records, are all examples of modern technology [1]. Because of its ability to link individuals and groups in a variety of industries, cyberspace is currently seen as a huge leap forward in comparison to other major sectors in the world of progress. Cyberspace is now ingrained in every aspect of our lives [2].

Most businesses nowadays place a high premium on security to safeguard their Information and Communication Technology (ICT) business environments from cyberattacks. Furthermore, due to financial and criminal objectives, many economic businesses and cultural/educational institutions are vulnerable to a variety of threats (e.g., viruses, worms, Trojan horses, and spyware), resulting in a tarnished company reputation, large financial losses, and the leakage of personal data belonging to users or consumers [3]. For all of these reasons, it has become vital for each organization to have certain security policies to safeguard their operations [4]. Security is defined as the state of not being endangered by any sorts of risks, including those that are physical, psychological, monetary, emotional, and so on [5]. Communities, nations, and businesses all have a stake in security. At the national level, there is a larger influence on security [6]. As a result, security is quickly becoming one of the most important aspects in the success of any corporation or business, whether public or private [7]. Therefore, cybersecurity (CS) is required in the form of a collection of security policies that specify what represents appropriate and inappropriate actions of users in relation to the secure handling of information assets [8]. Establishing a set of information security policies and procedures is as important as having technical solutions for CS [9]. In this paper, security policies refer to tools, regulations, rules, procedures, ideas, management techniques, and best practices. Encryption, error-checking techniques, preventive systems, and detecting instructional tools may all be used to combat many security risks [10]. Such precautions are taken to, firstly, ensure that data are only accessible by authorized users, which is known as “confidentiality”; second, to ensure that data are true and accurate and that malicious software does not deface data, which is known as “integrity”; and third, “availability” refers to ensuring that all network devices are available to the real user [11].

The effective implementation of CS policies is coupled with challenges in areas such as choosing the most appropriate controls, understanding the organizational needs, dissemination and management of policies, awareness training, and monitoring user’s behavior [8]. The relevance and applicability of CS policies differ across multiple industries. It is imperative to compare and choose the most relevant CS policies depending on the context of a specific enterprise. Hence, there is a need to compare the CS policies from various industries to develop a deeper understanding before building a CS policies framework. To fulfill this need, this study compares the security practices of five different types of businesses: healthcare, finance, aviation, education and learning, and e-commerce. The selection of these industries was based on their wide distribution and different levels of security among each of them. There are several resources for developing CS policies, but little is known about common CS policies for various businesses. As a result, to close this gap, this study tackles the following research questions:

RQ1: What are the aspects of cybersecurity that different enterprises have in common?
RQ2: How are the cybersecurity aspects used among different enterprises?

This study performs a review based on several scientific papers found in academic databases. There are also reports from various corporate websites that include statistics on information and policies. The major contributions of the paper are as follows. First, we identified 10 common CS policy aspects for the above enterprises, namely, privacy, website, Cloud computing, email, physical, network, information, access control, data retention, and data protection [12]. Second, a comparison of these aspects among these enterprises was discussed. Finally, the future research directions of CS policies were discussed. The following is how the rest of the paper is structured: Section 2 covers the research background and related work, Section 3 discusses CS policies, Section 4 discusses the findings and future directions, and Section 5 concludes the article.

## 2. Background and Related Work

This section presents the existing work and literature on the research topic and reviews the advancements and limitations of the main subjects linked to the research. First, it develops the research context by outlining the five most typical sectors that provide Internet services. Following that, it summarizes the existing knowledge by providing an overview of what the scholarly literature states on the topic at hand.

### 2.1. Research Context

In terms of access to information and news, as well as aiding various day-to-day living concerns and chores such as bill paying, purchasing, and shopping, the Internet has dramatically transformed the reality of communities. Healthcare, banking, aviation, education & learning, and e-commerce are among the industries covered in this area.

Healthcare: E-healthcare is ushering in a major change in the way technology is used in electronic health records. The coronavirus pandemic has increased the criticality of telehealth technology as a key element of modern and sustainable medicine [13]. Many individuals have benefited from significant health advances as a result of technological breakthroughs in recent decades, including clinical portals and patient portals [14]. Patients may view their personal health information at any time and from any location. By providing telemedicine services, e-healthcare has become the best option for reaching far-flung areas. Medical health websites enable patients to receive reliable and accurate information from a variety of sources [15]. Patients may use e-healthcare applications to manage their health information, make appointments with their doctors, and obtain test results promptly. All of these services need advanced CS techniques and procedures [16].

Finance: The financial industry and many economic sectors throughout the world rely heavily on CS. Customers may use e-banking to create accounts, manage those accounts over time, conduct financial transactions, access various financing sources, check out other accounts, and pay monthly payments from anywhere and at any time that is convenient for them [17]. Customers may make financial transactions from the comfort of their own homes rather than visiting a bank location. E-banking services, such as ATMs and electronic transactions, are also available to anybody. Users may pay utility bills via their mobile phones and smartphones, whether they are at home or work [18]. Moreover, customers may monitor and perform transactions by going onto their bank’s website, entering their username and password, and continuing without having to go to the bank [19].

Education and learning: In most educational institutions, online learning has become a common practice. Most colleges have embraced online education in recent years, using a variety of platforms and technologies such as Zoom, MS Team, instant messaging, and so on [20]. Learners may interact in real-time from their mobile devices over long distances using online learning [21]. Other learning aids include training materials and wizard performance assistance software, which may be used by students [22]. Digital libraries, such as those run by the Institute of Electrical and Electronics Engineers, the Association for Computing Machinery, and the Web of Science, are also available thanks to advanced technology [23].

Aviation: The aviation technology industry has been evolving and growing at a rapid pace. The e-aviation business offers a variety of electronic services. To begin with, software programs assist passengers with ticket reservations by providing a substitute, such as the e-tickets system, which allows travelers to purchase tickets via the Internet from anywhere, at any time [24]. Requests for travel timetables and itineraries can also be made online. Second, there are a variety of programs that provide services to passengers and are simple to install on a computer. Software tools, for example, allow users to search for the cheapest tickets and check in to flights from home or anyplace else [25]. Similarly, electronic checklists, inexpensive aircraft charts and plates, simple parking services, and aviation weather services are all made possible by Internet technology. Finally, contemporary facilities for training pilots have been developed as a result of enhanced technology, such as digital aviation training software, virtual classrooms, computer-based training equipment, and simulations [26].

Electronic commerce: E-commerce is a new, clever technique for conducting business through the Internet. Today, anybody from anywhere across the globe may easily purchase and sell products and services through the Internet [27]. E-commerce also offers a variety of services, such as credit card-based electronic payments, commonly known as “digital money”. E-commerce has had a significant influence on nearly every continent’s economy. Asia has grown to be the world’s largest online market. At the end of 2005, Internet transactions in the Asia Pacific and Latin America totaled more than USD 5 trillion [28]. Digital payments are expected to reach USD 6.6 trillion in 2021 [29].

### 2.2. Literature Review

We identified a lot of studies in the fields of CS and ICT security policies when we reviewed the literature due to the topic’s importance in everyday life. This paper distinguishes between information security and computer science. Information security is the protection of data from harm; CS, on the other hand, has a broader meaning, aiming to safeguard not just data but also people who use cyberspace and their assets, as well as cyberenvironments’ defensive communication technologies, from hackers [12,30]. The following sections cover CS and information security literature.

#### 2.2.1. Cybersecurity Policies

Weiss and Biermann [5] reviewed and compared worldwide legal security and privacy regulations utilized in areas such as banking, healthcare, and education. London [31] looked at many aspects of information technology as well as the implications of a lack of knowledge in the sector. The authors looked at data protection, data security, and information privacy laws in five countries, as well as what privacy-enhancing technologies are available, especially under US laws such as the Health Insurance Portability and Accountability Act, Gramm–Leach–Bililey Act, and the Family Educational Rights and Privacy Act. Yoo [32] classified personal identity information violations into medical, financial, and socially acceptable categories, determining which privacy protection laws, such as the Health Insurance Portability and Accountability Act, Gramm–Leach–Billey Act, and Economic and Clinical Health (“HITECH”) Act, are required for each. The author also advocated for the establishment of compensation mechanisms for data acquisition and misuse that occurs without the permission of persons. Although establishing database protection compensation is difficult, the author reported that such rules should exist for private businesses. In addition, the author discussed the significance of data protection awareness. Liu et al. [33], to maintain privacy and security on the Internet, suggested utilizing authentication and access control measures. Alotaibi et al. [8] highlighted the challenges organizations face in the adoption of information security policies. Persadha et al. [34] conducts a comparative study on a delegation plan of responsibilities in cyber-related roles, and categorized them among three different countries.

Different website security policies were examined by Saiedian and Broyles [35]. They also suggested that developers use various security measures, such as scanning tools and services. User Specified Content Security Policy, a Firefox addon to defend websites against content injection attacks, was suggested by Patil et al. [36]. They also advocated for the adoption of user-content security rules, which would allow developers and users to create such papers for the website. Web security may be guaranteed by restricting third-party access while allowing trusted individuals access and imposing access control to provide cryptographic server identification [37]. Martins et al. [38] discussed the possible risks of employing Information ICT in the banking industry, as well as the critical role that ICT security regulations play in mitigating such risks. The authors discussed certain key security measures, such as using a trustworthy and fast Internet connection to conduct financial transactions, using secure communication to maintain consumer confidence, and enabling ICT monitoring. The author also mentioned the need for technical assistance, as well as the need for privacy policies in protecting personal information [39]. Abomhara and Kien [40] evaluated IoT device security and the sorts of risks that exist in the IoT context. Security policy, according to the authors, includes not just the security of services, data, and information, but also the security of physical equipment. The authors also recommended security measures such as access control, authentication, and identity management to combat threats [41].

The challenges of data security and privacy protection on Cloud computing were discussed by Sun [42]. The authors described Cloud security as the combination of computers, networks, and information, and explained privacy regulations and how they differ between nations. They also noted that sensitive and nonsensitive data should be segregated, with the former being encrypted. Security requirements in Cloud computing infrastructure were classified into three levels by Zissis and Lekkas [43]: Application level (e.g., data privacy and access control mechanisms), virtual level (e.g., application security and Cloud management control), and a physical level (e.g., application security and Cloud management control) (e.g., hardware security and network resources protection). They stated that the level of trust in Cloud computing is determined by the model used, as well as physical and program-related security regulations. Second, secrecy is accomplished by allowing only authorized parties access to protected data. Subashini and Kavitha [44] highlighted how data leakage in companies may be avoided by employing techniques such as data–user segregation, data backup, secure encrypted storage, and restricting access to particular data to only allowed individuals, therefore limiting access to only authorized workers in the firm. The authors also recommended authentication and authorization to govern data access. To assure security, the authors emphasized the usage of encryption methods for data transit and storage. The authors stress the need of enacting data protection legislation in government [45]. Cotropia et al. [46] looked at the data retention rules that are used to govern the secure storage and retrieval of electronic data in computer systems and recordable media in electronic information systems. The author suggested a software module for media management components that is responsible for data storage, archiving, and recovery, as well as keeping multiple copies of data and information systems.

Daniel [47] investigated Cloud computing issues in educational firms, such as data security and sensitive data threats. The authors discuss contemporary security methods for ensuring data, service, and infrastructure confidentiality, integrity, and availability, including federated identity management, data masking, firewalls, encryption, and key management. In addition, access control regulations in universities prevent illegal access to data in institutions other than by students, professors, and staff. Bandara et al. [22] stressed the importance of putting in place information security policies at universities to protect resources, in addition to personal, sensitive student data, and to protect private learner data, financial and bank-related course fees and payments, and any other potentially sensitive data against attack or abuse [39]. To avoid data tampering and maintain the integrity of data, Kalpana and Singaraju [48] recommended utilizing RSA algorithms, secure methods to assure privacy and confidentiality for permitted access, and secure virtual memory and storage in Cloud service providers.

Goyal et al. [49] reviewed the literature on mobile security problems, strategic, legal, and ethical issues in mobile banking. Customers should be aware of trustworthy mobile banking, according to the authors. Additionally, the significance of safeguarding client data by allowing only authorized customers to process associated financial information [49]. Other security options proposed by the authors include encryption, PIN authentication, firewalls, and third-party services for added security protection. These security principles, according to the authors, should be used to control secure mobile banking services between customers [49]. Yildirim et al. [50] examined e-commerce security practices among small- and medium-sized businesses in Turkey. Information security policies, rules involving access control that commit users to utilize privileges to access particular levels inside business systems, network security policies, and physical security policies, were all categorized by the authors into different groupings. Wu et al. [51] investigated the importance of security policies in managing the e-commerce process and protecting consumers’ personal information. To develop consumer trust, this function is expanded in an e-commerce website. The Security Federal Trade Commission creates these policies [51]. The authors also stressed the significance of consumer knowledge in preventing the abuse or breach of personal information. It is suggested that a trusted third party be used to obtain authorization to release client information, as well as the use of secure email [52].

McCallie et al. [53] looked into security in aviation systems, particularly in autonomous and reliant surveillance-broadcast systems that use unencrypted data to determine aircraft positions, which necessitate security measures. The authors stressed the necessity of security for the automatic dependent surveillance-broadcast components and all electronic aviation equipment, as well as physical security in places such as the aviation station. Several security procedures were proposed by Sampigethaya et al. [54] to safeguard airline administrative and passenger services, flight operations, and air traffic management from potential cyberattacks. Firewalls, monitoring systems, fiber cables, power lines, and gigabit copper are among the safeguards used to ensure dependable and secure communications. Other security standards include utilizing tamper-proof logging in all aircraft systems to assure passenger and crew authentication. The usage of secure separation between flight essential data and other forms of information is also recommended. As a result, adhering to security policies is critical for aviation safety [54].

#### 2.2.2. Information Security Policies

Panda [55] defined network security as “information security for all information transmission networks,” and classified it into three categories: hardware, software, and information. They suggested a variety of security mechanisms, including data encryption, access control, unauthorized users being blocked by a trusted third party, authentication techniques such as identification names and passwords, network traffic being monitored via firewalls, Intrusion Detection systems (IDs) and prevention systems, and antivirus software. Subramanian and Kumar [56] presented an information policy for various organizations, including updates for antivirus software, data and router backups, unwanted file cleanup, and mail backup and maintenance.

Information security policies and increasing information security compliance was emphasized by Crossler et al. [57]. The relevance of behavioral information security, as defined by the author, is concerned with users’ behavior in protecting information through the use of antimalware software, data backup, and secure wireless networks. The authors also mentioned data collecting and measurement solutions that are available to provide exclusive access to authorized individuals alone [58]. Stahl et al. [59] investigated the significance of information security policies, and looked at examples of them in the healthcare industry. They also highlighted the company’s accountability policy, which includes disciplinary action for any data breach, as well as the fact that any data breach would be treated as a criminal offense, and workers’ access rights may be revoked as a result of such violations.

Susanto and Almunawar [60] analyzed information security awareness and its influence on corporate business operations. All employee responsibilities to safeguard physical and private information, as well as computer devices, were recommended by the authors. The authors discussed standards of information security such as the “ISO 27001” security system, which is meant to protect the availability, confidentiality, and integrity of information. The authors mentioned password rules, the use of two-factor authentication, and security against physical access to information-technology infrastructure as security measures to guard against information-targeted assaults [37]. Bilbao et al. [61] reviewed big data, data sharing, safeguarding private data and trust-sharing network data, utilizing biometrics to provide secure identification, and ensuring trust network agreements through a third party.

## 3. Results

### 3.1. RQ1: Cybersecurity Policies Taxonomy

The Internet has become the new reality, and people have devoted their lives to embracing it, to the point that the issue is being treated as a human rights issue in certain nations. However, while cyberspace may include helpful domains, it also has security and privacy concerns [62]. Because many of these contemporary, sophisticated cyberthreats and attacks may readily bypass security and privacy, cyberspace is all but unsafe. The following are some of these threats [16,63,64,65]:

- Viruses: Viruses are computer programs or files that may move from one computer to another, infecting the target computers and allowing the infection to propagate further through the Internet;
- Worms: Worms, by design, are comparable to viruses and are classified as a viral subclass. The worm travels from computer to computer, but unlike viruses, it may travel without the need for human intervention;
- Trojan Horse: A Trojan is a software that looks to perform a helpful purpose but actually performs a secret function that poses a security risk;
- Spyware: Spyware is a type of harmful software that infiltrates computer systems to collect personal information from users. It usually comes through compromised computers or websites;
- Impersonation: In an impersonation attack, the attacker poses as a real user or server and provides false or harmful services to genuine users;
- Man-in-the-Middle: The attacker in a Man-in-the-Middle attack surreptitiously transmits and maybe changes messages between communicators without disclosing them to legitimate users;
- Denial of Service Attack (DoS): A DoS, also known as a distributed DoS (DDoS) attack, is a type of website assault in which an attacker floods victim’s computer with a huge amount of data packets. A DDoS occurs when attackers obtain unauthorized access to a large number of computers on the Internet, including the target systems.

CS has a variety of objectives in any business, such as: [15,62,66,67]: (1) It helps businesses meet their objectives and fulfill their goals by supporting and implementing their needs. (2) Its goal is to guarantee that there are no more bad guys, and to determine what security steps companies should take to avoid cyberthreats. (3) It influences the amount of staff discipline. (4) Furthermore, CS strives to deliver secure services. (5) In addition to other responsibilities, it ensures the security of information technology resources. (6) For businesses, CS determines the areas of security vulnerability. (7) Another goal of CS is to reduce the possibility of cyberattacks, as well as to create a secure environment that is unfavorable to cybercriminals. There are also business criteria that define which CS must be used. For example, if consumers must use the Internet, if customers must access specific data, or if customers must utilize or save data. In general, any CS has a goal (i.e., what is the policy’s aim? Why is this policy needed?), scope (i.e., What are the activities or assets of the organization to which this policy will be applied? What IT resources are available? What hardware, software, information, or people, will be used to implement this policy? Who should follow this policy?), and compliance (i.e., how is the policy enforced? What are the consequences of failing to comply with the law?).

**Figure 1:**
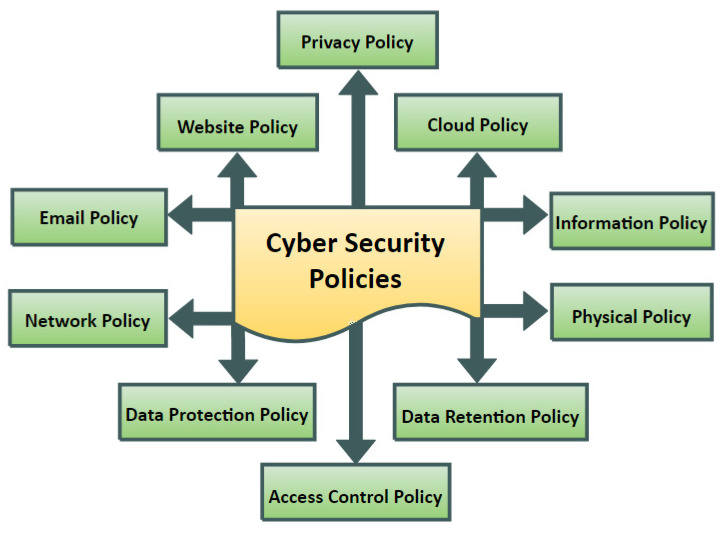
Cybersecurity policies taxonomy.

There is little knowledge regarding common features of CS policies for different types of businesses, and current CS policy research does not look into what the best CS policies are for businesses. Furthermore, in recent years, an increase in cyberattacks on various businesses has resulted in catastrophic losses for businesses. As a result, it has become clear that new methods for locating the finest and most secure solutions for this purpose are required. The current study’s advantages are in tackling more significant security policies in a variety of businesses to provide a comprehensive picture of the secure electronic community in these sectors. This research identified 10 common CS aspects, as shown in Figure 1, from the literature: privacy, website, Cloud, email, physical, network, information, access control, data retention, and data protection. Table A1 (Appendix A) shows past surveys, and illustrates how this publication varies from previous research. The peculiarity of this work is that it synthesizes all papers on cybersecurity challenges in the specified scenarios in order to offer better knowledge and extrapolation of the study topic.

#### 3.1.1. Privacy Policy

The objective of this policy is to model the correct use of sensitive personal data, such as medical data, biometric data, and financial data, in terms of the agreed-upon rationale for utilizing these sensitive personal data and to protect them from violations [68]. Accordingly, it prevents the disclosure, use, access, collection, transfer, and exchange of sensitive personal data without the knowledge of persons, by tightening control via user consent, or a responsibility to keep data safe by a data-controlling organization’s trustworthy administration [69]. In addition, a privacy policy protects both intellectual property and personal data [70]. This policy complies with human rights to privacy and the protection of sensitive data. Furthermore, it establishes penalties on anybody who violates the privacy of consumers’ data in a commercial setting [71]. Therefore, privacy laws, according to US Federal Privacy Legislation, are divided into four categories: protecting customer’s data, protecting children’s data, protecting patient’s data, and protecting credit card information [72].

#### 3.1.2. Website Security Policy

The correct usage of online applications and services is defined by this policy. The goal is to determine the level of security and identify vulnerabilities in websites [73]. It also serves to safeguard critical client information from harmful scripts on other pages [73]. This is to avoid web application assaults such as scripting, that injects programs into data-driven applications [74]. In this regard, the content security policy outlines the typical techniques for loading material onto websites. Furthermore, the same-origin policy allows data on a second webpage with the same origin as the first to be accessed. Any website’s browser actions are recognized and tracked by a third-party web-tracking policy. Consent-based cookies are how this is accomplished [38]. Access control in the form of authorization rights is used across the website security policy to secure personal data, such as data found on social media platforms [75].

#### 3.1.3. Cloud Computing Security Policy

The goal of this policy is to guarantee that Cloud services meet security standards as well as legal and regulatory obligations [76]. A Cloud computing security policy is a document prepared by top management for the whole Cloud system to notify all workers and important external parties. The first Cloud security policy addresses all aspects of security, including access control, data storage, and encryption. Second, it addresses network issues such as transmission security. The third part is computer security. To enable secure data exchange and interactions, the Cloud employs a trusted third-party policy [77].

#### 3.1.4. Email Security Policy

This policy is divided into three parts [46,78,79]: First utilizing emails as per user recommendations. This policy aims at clarifying what constitutes appropriate email usage, and to educate all employees on what constitutes acceptable and unacceptable email usage. Some of these recommendations are to use emails solely for business purposes, protect data and attachments sent via email, as well as any business information included in them, to not send disruptive or offensive communications by email, and to not send personal messages using the company’s title. Second, email security policies serve as a guide for administrators in businesses. The goal of this policy is to keep track of all message traffic and content, as well as to archive and examine user emails. Third, there is the utilization of encrypted communications and digital signatures to prevent spam messages when communicating through email.

#### 3.1.5. Physical Security Policy

The goal of this policy is to secure the organization’s assets, resources, equipment, hardware, and facilities against unauthorized people damaging or stealing them [5]. Furthermore, this policy uses access control measures to prohibit unauthorized individuals from accessing the assets of the business. It also attempts to safeguard an organization’s information systems, physical systems, human assets, and those who interact with these assets [12]. Another objective is to secure the infrastructure of the cyber-physical through implementing detection systems and monitoring measures, for instance. Moreover, clear screen and clean desk policy, acceptable use of physical assets and other techniques, and, more importantly, to educate end-users on how to protect their computers and other cyber-assets from loss or unauthorized access by using stronger passwords and following the guidelines of policies [80].

#### 3.1.6. Network Security Policy

The security of network components, connections, and contents is the focus of this policy. It also attempts to guarantee that the network is trustworthy and that users are informed of what is acceptable and what is not [51]. This is conducted to secure computer networks and communication equipment including routers, switches, and servers, as well as information and any service transfers that take place across these networks [81]. Special hardware components, such as detection systems and firewalls, are used in this strategy, to safeguard networks from unauthorized access or unintentional change. Furthermore, it focuses on defensive techniques such as encryption, which is used to secure data transferred over the Internet [24]. Another goal of this strategy is to provide secure network administration [24].

#### 3.1.7. Information Security Policy

Information resources in any company are protected by rules [57]. The goal of this policy is to establish guidelines for organizations to follow to safeguard all physical and digital assets against illegal access, copying, modification, disclosure, destruction, and transfer to third parties for personal gain [82]. Furthermore, this policy protects the digital storage of all companies’ data. The confidentiality, integrity, and availability of such resources are all ensured by these rules. This policy also safeguards information within the organization’s networks [12]. Furthermore, by offering best-practice standards for corporate workers to follow, these policies empower employees to engage in the safeguarding of the organization’s critical information [83]. In addition, to reduce security risks in companies, information security policies describe the management’s attitude and characteristics.

#### 3.1.8. Access Control Policy

These rules protect physical resources, information systems, and IT resources against unauthorized access by identifying, authenticating, authorizing, and monitoring who has access to them [43]. In this regard, several steps are implemented. Restriction mechanisms, access control, permission mechanisms, and authorization are examples of systems that translate users’ access requests to govern the usage, entrance, and consumption of an organization’s resources or network services [10]. Moreover, the access control policy restricts actions to just authorized individuals and controls all access to the organization’s systems. Some organizations use authentication to control access, while others use both authentication and authorization to manage access to their resources, depending on the structure of the organization, the degree of data sensitivity of organization documents, and the level of such sensitivity allowed for users to access [37].

#### 3.1.9. Data-Retention Policy

This policy specifies which data should be retained, how long it should be kept, and in what format it should be stored [84]. The goal of this policy is to protect vital information by storing it in encrypted data backups for a certain amount of time [17]. Additionally, this policy allows for frequent archiving to allow personnel within the organization to readily access and delete any files that are no longer needed, as well as the encryption key that encrypts the data. Accordingly, the US Data Retention Act only enables Internet service providers to store data for two years [17]. While some institutions are allowed to store such data under the Health Information Privacy and Accountability Act, others are not [85].

#### 3.1.10. Data-Protection Policy

The goal of this policy is to protect the processing and management of personal data [86]. This policy guarantees that third-party data are collected, utilized, shared, stored, transported, and sent securely, to use the data for needed and defined reasons [87]. It also establishes the anticipated behavior of employees when dealing with such material [20]. Moreover, this policy describes how businesses should handle consumer data and raises user awareness to prevent data loss [67]. Because data security is one of the most significant elements that affect a company’s reputation in business, depending on the kind of data and the level of sensitivity, each organization should categorize data that needs to be monitored [47].

### 3.2. RQ2: Comparison of Cybersecurity Policies among Selected Enterprises

In this part, we analyze the common CS aspects of the five companies that form a domain in business–consumer transactions (healthcare, finance, aviation, education, and e-commerce). Table 1 summarizes the differences in the level of different CS aspects among these five enterprises. Each color reflects the relevance of a certain policy. The reason for the disparity in the importance of CS is due to the types of data and information handled by businesses, as well as the interests of each business, its size, and its security requirements.

#### 3.2.1. Privacy Policy

As the comparison matrix demonstrates, any firm in the healthcare, financial, or educational sectors should have privacy policies in place to protect sensitive personal information such as health and financial data, as well as data acquired at universities. The HIPAA Privacy Act, the GLB Act, and the FERPA Act should all be followed by these three organizations. Furthermore, to secure consumers’ credit card information, e-commerce industries should adhere to the Fair Credit Reporting Act. Using encryption or authentication techniques to establish privacy and maintain data confidentiality is highly backed by numerous studies. According to the findings, there should be privacy regulations in place to protect passenger data. The Lowa Bank company, for example, safeguards sensitive customer data by using an encryption method that encrypts all financial client data and ensures consumer privacy. Table 2 summarizes the role of policy privacy among the five enterprises.

Healthcare: The level of privacy in healthcare is very high. Patients have the right to privacy with this information in healthcare [71]. Because of the health aspect of this data and their look, wearable personal health monitor devices are another example [88]. Furthermore, in networks that connect hospitals and patients, privacy is essential in end-to-end applications. Additionally, this is required in the health Cloud, which necessitates the use of secure key exchanges for the safe transfer of personal data, and as a result, the ability to manage this flow by designating who has access to such data [15,89].

Finance: The Gramm–Leach–Bliley (GLB) Privacy Act should be followed by banking sectors to prevent the exchange, misuse, collection, or disclosure of financial information without the customer’s consent [90]. Customers have the right to view and update their financial information under the GLB privacy legislation. The restriction of revealing clients’ account details through marketing electronic mailings is another GLB privacy policy [71]. According to these privacy standards, all financial institutions must use all security measures, such as encryption, to protect data confidentiality, as well as access control processes to ensure authorization and authentication [91].

Education: The security of sensitive student information is one of the most significant concerns for educational institutions; as a result, many colleges and schools have embraced the Family Rights and Privacy Act, which governs access to and use of personally identifiable information about students [93]. This act prohibits the public disclosure, distribution, or discussion of a student’s grades without their permission. Student ID numbers, not student names, are required to declare grades for this reason. This law also protects reports regarding a student’s psychiatric condition, handicap, and personal or academic conduct [47,92].

Aviation: One of the most significant concerns is the protection and confidentiality of passenger personal information, such as passenger names, residences, birth dates, contact information, and trip data [94]. Privacy rules include all sensitive health and religious information, as well as information, that is shown on body-scanning equipment at airports. In terms of passenger rights, the protection of this information is regarded as a top priority [95]. Another airline privacy regulation involves encrypting passengers’ digital information profiles to prevent identity exposure in aviation databases [25,96].

Electronic commerce: During the procedures of online transactions, the privacy and security of customer data are important to ensure that personal information is only accessible by authorized users [28]. Furthermore, privacy policies in e-commerce define who has access to information, how it should be protected, and the level of control that customers have over their own data [98]. It also includes additional private information such as credit card numbers [86], in addition to personally identifying information. In this case, customer privacy protection includes preventing the disclosure of customers’ identities through the use of customer anonymity, as well as prohibiting the exposure of their transactions by a variety of privacy violations such as profiling, investigations, and discrimination, among others [88,97].

#### 3.2.2. Website Security Policy

In the financial, educational, and e-commerce sectors, website security policy is a crucial problem regarding creating consumer confidence, preventing cross-site scripting and clickjacking assaults that insert harmful code into websites, and dealing with same-origin regulations and content security measures. One of the most significant recommendations in this study is that a formal act is enacted to implement website security regulations to examine the security of e-commerce web applications [73]. Lowa Bank uses secure log-out sessions on their website as one of their website security practices. For example, after 10 minutes, a customer’s account will be immediately closed [91]. Table 3 summarizes the role of website security policy among the five enterprises.

Healthcare: Websites in the healthcare industry should safeguard patients’ data. Furthermore, to secure patients’ data, health website server providers must adhere to healthcare regulations [4]. On the associated websites, it should also provide secure health services and applications such as utilizing Microsoft Health Vault and Google Health to manage health information securely on these platforms [15].

Finance: On their websites and through financial web applications, most financial firms offer online services. As a result, these utilities must be subjected to stringent privacy and security regulations, as usually linked to computer systems that keep track of sensitive customer data. It is critical to examine these applications on the banks’ websites to ensure that they are immune to various vulnerabilities [99]. Another security strategy in online financial websites is the use of strong passwords and authentications to log in, to provide more security and authenticity [42].

Education: To prevent security risks such as cross-site scripting, secure web applications and resources are necessary on the local website. Authentication and authorization procedures guarantee that resources on university websites are accessible in a secure manner. These websites will have auditing and monitoring measures in place to track visitor behavior. On the website, cryptography is used to secure pupils’ private data [99]. Moreover, code review and inspection techniques should be used to evaluate and defend university websites against vulnerabilities, such as search engines that are put to the test using Internet security mining tools [47]. Academic databases may be monitored for security, while testing is necessary to examine the security of academic websites by verifying data and analyzing web addresses for threats or malicious code injections [74].

Aviation: To communicate with passengers and provide secure airline payment services, only secure websites should be utilized [100]. Customers may purchase electronic tickets and check-in online, as well as secure electronic seat selection, using the secure website [74].

Electronic commerce: Using safe websites in the e-commerce industry is one of the most important aspects of ensuring e-commerce’s an excellent reputation for integrity, as well as gaining customers’ confidence [73]. As a result, comprehensive security measures on related websites are required, one of which is to ensure the confidentiality of sensitive data by employing encryption mechanisms during online transactions. Another option is to employ secure socket layer certificates on e-commerce websites as an additional security precaution for consumer logins to pages and sign-ins and sign-outs. Malware and virus programs cannot infiltrate Internet companies if extra protection layers such as security solutions, for example, firewalls, are used [73]. Furthermore, for e-commerce websites, dependable secure shopping cart software is strongly recommended [101]. Extra levels of protection, such as protected search queries or login boxes, can always assist to avoid malicious code injections and cross-site scripting attacks on e-commerce websites [102].

#### 3.2.3. Cloud Computing Security Policy

The majority of today’s businesses require security and privacy in their Cloud computing settings. E-commerce, airlines, banking, universities, and colleges are among these businesses. The health industry should adhere to HIPAA privacy laws in its Clouds because the kind of data handled in these businesses, which includes health information, financial information, credit card information, and intellectual property, necessitates a higher level of privacy and transparency. Furthermore, the Federal Information Security Management Act is key legislation that addresses security concerns in commercial Cloud computing. For commercial Cloud computing, formal privacy and security legislation is necessary. To safeguard the enterprise’s Cloud server, Lowa Bank utilizes a secure location for its data center and a secure banking server as one of its Cloud security policies. Table 4 summarizes the role of Cloud computing security policy among the five enterprises.

Healthcare: It is critical to provide a high-security level for the healthcare Cloud, and any business should expect a secure connection between healthcare providers and patients and a high-security level of data storage [1]. Furthermore, when such activities are supervised by healthcare experts and only patient or physician authentication procedures are employed, the confidentiality of electronic health data is achievable [103]. Encrypting data before storage in segmented ways and between two separate and independent portions, as well as encrypting data before storage in the health Cloud, should be supplied in tandem to secure sensitive health data [103].

Finance: Cloud security plays a critical role in financial firms since it is essential to have the proper solutions in place to protect client information by utilizing secure Cloud applications and data transfers [1]. Cloud security regulations protect data from being leaked or damaged by preventing data and programs from being viewed by other applications [104]. In addition, access control mechanisms must be used to limit the availability of financial Cloud services to other organizations and secure data stored on the firm’s server providers [105].

Education: To safeguard university Cloud computing and ensure student data privacy, all institutions shall adhere to security and privacy rules based on the Family Rights and Privacy Laws. The following are some of the security measures in place: First, they must utilize authorization and authentication methods to access university Cloud or student data, to safeguard student sensitive data access, storage, transfer, and exchange via university Cloud [10]. Second, data transferred over the university Cloud must be encrypted, and IDs and prevention technologies must be used to monitor network traffic in the Cloud. Third, failure to follow Cloud policies should result in disciplinary action [83]. In Cloud computing settings, data location must be transparent, and detecting systems must be deployed with preventative and monitoring equipment, as well as antivirus software filters [10].

Aviation: The security of Cloud computing, which is used to manage very large amounts of data in complicated aviation environments and offer secure services over the Internet between aircraft and ground operations, is one of the most pressing concerns in the aviation sector. Encryption of data is one security approach in this regard [106]. Moreover, access control techniques must be used to protect any data in the Cloud environment from unwanted access and to ensure that backup data are stored safely. Furthermore, digital signatures can verify the validity of communications or documents sent via the Internet or in the air [107].

Electronic commerce: Cloud computing for e-commerce security and protection measures safeguard the firm from DDoS assaults, ensuring Internet availability and secure online transactions and communications [102]. To maintain authority, authenticity, integrity, nonrepudiation, and confidentiality of information, all technological architecture, including hardware such as servers and any other IT resources and software of the Cloud, must be protected. E-commerce may also benefit from the use of secure distributed Cloud systems. Cloud computing will take place for dependable services while also ensuring the security of customers’ data [108].

#### 3.2.4. Email Security Policy

According to the findings, every business should follow and adopt the CAN-SPAM Act principles to govern commercial email and avoid spam communications. The healthcare industry, on the other hand, should adhere to the HIPAA Act to secure patients’ electronic mail. To guarantee safe communication between students and universities, colleges and universities should also utilize filtering spam techniques to avoid any unethical communications and distinguish phishing and spam emails from legal emails [109]. The findings show that the CAN-SPAM Act is a superb security measure for ensuring secure communication between clients and businesses. The email security rules of Lowa bank include forbidding the sending of any financial information through email and informing all customers not to respond to any email claiming to be from the bank without first contacting the bank and confirming that the email is from the bank. Table 5 summarizes the role of email security policy among the five enterprises.

Healthcare: To safeguard patient data and staff’ credentials from assaults, it is critical to utilize secure emails in the healthcare industry [110]. The security of electronic health information is ensured via a secure connection between patients and hospitals. An example of an email security law is the Health Information Technology for Economic and Clinical Health Act that ensures the protection of personal health information in emails or data transfer. In addition, emails should contain protective marking based on the sensitivity of information included. If email security in health sectors and hospitals is not adhered to, there should be sanctions and responsibility [111].

Finance: When consumers and financial firms communicate through email, they should use digital signatures or install mail filters to protect themselves from phishing messages or spam letters [112]. All financial firms should make it illegal to communicate their clients’ financial information through emails, such as credit card numbers or online account numbers [101]. Furthermore, these businesses should implement encryption technologies to provide safe email contacts with consumers [104].

Education: Maintaining safe communication between students and teachers is one of the most important goals for academic institutions and universities. Because students’ information, such as passwords, may be stolen, email security regulations guarantee that spear-phishing communications are avoided, as well as suspicious links and attachments received from fake addresses [79]. Furthermore, the security of students’ email systems protects their inboxes from spam by employing spam-filtering algorithms [113].

Aviation: In the aviation sector, email security is critical since it is linked to airline services and consumers; it must adhere to email security measures such as preventing undesired spam and links that contain dangerous files by employing software filters [114]. Furthermore, while sending payment for airline bills through email, it is critical to choose a reputable service [25,113].

Electronic commerce: Sensitive data and private customer information are rarely sent by email, and responding to emails necessitates updating account bank information and credit card information [102]. Avoid clicking on questionable email attachments, links, or addresses; instead, make direct contact with online purchasing websites, and use email-scanning tools to avoid phishing messages and identity theft, as well as spam unwanted commercial emails. Furthermore, all confirmation messages and emails provided to consumers by e-commerce merchants or online shopping businesses must be printed and saved [115].

#### 3.2.5. Physical Security Policy

In comparison to other businesses, hospital and aviation systems have the highest priority in terms of protecting critical infrastructure and cyber-physical systems. Furthermore, not only the health and aviation industries but also financial institutions should give this issue of infrastructure protection top attention. According to the policy’s findings, there should be a formal physical security law to protect vital infrastructure in the health, financial, and aviation sectors, as well as sanctions for asset destruction. Lowa banks have implemented physical security practices such as employing antivirus and antispyware to safeguard software systems, as well as deploying digital locks and surveillance equipment to protect bank computers from illegal access or manipulation. Table 6 summarizes the role of physical security policy among the five enterprises.

Healthcare: It is critical to offer a secure environment for patients and medical personnel [59]. To safeguard health information from damage or loss, security measures should be in place to protect physical gadgets in healthcare as well as other organizational assets including emergency rooms, buildings, and medical equipment [116]. Data centers containing personal health information should be monitored, restricting unauthorized people from tailgating.

Finance: These policies prevent illegal entry to buildings and any type of physical hardware, as well as the removal of data from disabled systems used in financial institutions and banks [80]. This category includes the use of alarms and other control mechanisms to prevent damage and manipulation of communication devices, electronic devices, payment systems, and other financial utilities, as well as the robbery of Automated Teller Machines [17].

Education: Physical asset security is critical in educational firms because it protects school assets from outsiders and provides controls access to these assets [92]. This policy also covers the protection of networking equipment, servers, and cables. Certain security systems, such as detecting devices, locks, and doors can provide this level of protection in schools [80].

Aviation: Protecting key assets of aviation systems and airports, such as information systems in airports, airline buildings, airline networks, towers, and so on, requires the use of detecting systems and monitoring as well as preventative devices in the aviation industry [117]. Another security technique is to safeguard aviation buildings and other restricted areas at airports using video monitoring and fire-protection equipment [5]. Authorization and authentication techniques contribute significantly to the security of aviation operations systems [25].

Electronic commerce: One of the benefits of e-commerce is that it eliminates the need for actual businesses in these settings [52]. As a result, the requirement for security is restricted to Internet access devices, such as computers and mobile phones [115]. It should also solely use access control and authentication techniques to safeguard physical resources such as servers, switches, storage facilities, and firewalls from unwanted access [98].

#### 3.2.6. Network Security Policy

To assist enterprises and organizations in defending themselves against cyberattacks, The Protecting Cyber Networks Act should be followed and embraced by private companies, which allows them to exchange cyberthreat information while respecting network users’ privacy [118]. To secure their networks, these businesses employ network security measures such as firewalls, IDs and prevention systems, firewalls, and encryption techniques [119]. The findings demonstrate that contemporary network security rules should be implemented to defend any company from any outside threats. As an example, one of Lowa Bank’s network security strategies is to use numerous intrusion systems and firewalls to filter traffic and stop hostile outside entities. Furthermore, this bank complied with the secure socket layer to encrypt any data transmitted across its networks. Table 7 summarizes the role of network security policy among the five enterprises.

Healthcare: To provide safe and reliable communication while protecting patient privacy, it is critical to apply security policies in order to keep wireless medical sensor networks safe [120]. To secure eHealth, numerous security measures must be followed, including firewalls, antivirus programs to protect against Internet assaults, and access control [121].

Finance: To perform transactions, all financial firms now rely on open networks such as the Internet. As a result, network security measures such as firewalls and data encryption should be implemented in this sector to protect other internal and closed networks from cyberattacks [122]. According to Alenezi et al. [74], financial firms should mandate the usage of authentication mechanisms to safeguard their networks and implementing secure operating systems in these networks. Additionally, antivirus software must be installed on computer networks or servers [123].

Education: Employing firewall and IDs to safeguard institutional resources, research, and student data in educational institutions from unauthorized access and network exploitation or abuse [119]. Using encryption techniques, network security rules protect data or student records from being lost or damaged while being sent over networks [124]. Another advantage of these regulations is that they ensure stable Internet connections and protect against denial-of-service attacks [15].

Aviation: To safeguard aircraft private networks from suspicious Internet traffic, it is critical to deploy monitoring and prevention technologies as well as firewalls [127]. In addition, alert mechanisms must be operational to monitor network transactions or the deployment of a reverse proxy to prevent illegal access and safeguard airport networks and resources from cyberattacks [125]. Another network security strategy is to use secure cable connections and to encrypt data transferred over these networks [126]. Together with antivirus applications on computer networks, access control techniques can safeguard computer networks [37].

Electronic commerce: E-commerce security regulations also mandate the use of firewalls to protect e-business networks and prevent any breach or access to networks or vital financial information. Furthermore, authentication and authorization procedures must be in place to obtain access to network device operating systems [102]. Auditing network traffic will be aided by IDs and monitoring equipment [120].

#### 3.2.7. Information Security Policy

According to the findings, all businesses must have strong information security policies and strategic strategies. The ISO 17799 standard is a valuable tool for developing robust information security policies in a variety of settings, including universities, banks, and e-commerce. The Payment Card Industry Data Security Standard, which safeguards consumer information, should be used by the e-commerce and banking industries. There is also evidence that colleges should adhere to the IT Infrastructure Library requirements, as well as the necessity of adopting BS 7799 and ISO27001 information security standards as appropriate standards in the e-commerce industry. The findings also suggest that there should be adequate information security procedures in place to secure the information of the company. Leicester University is an example of a company that follows the ISO 27,001 standard framework to safeguard its information and information system [22]. Table 8 summarizes the role of information security policy among the five enterprises.

Healthcare: Information and information technology in the healthcare sector, including but not limited to health information, statistical research data and medical knowledge, appointment scheduling, and health monitoring, should be protected [15]. Three security criteria must be protected: telecare medical information systems must be protected to provide dependable and convenient E-healthcare, encryption must be used to maintain confidentiality, and digital signatures must be used to assure integrity [128].

Finance: Because these sectors deal with valuable assets, information security policy in financial firms is a crucial part of the issue [130]. Information and information systems must be safeguarded at all stages of the banking system, since information is seen as a significant asset in this sector, and its loss can result in serious financial and reputational damages [4,129].

Education: In educational institutions, information security is the top security issue [22]. The objective is to save any information that supports the university’s operations and helps it achieve its mission and vision [132]. Information assets include data about employees and students, professors, payments, teaching and learning materials, money, and scientific research, as well as the systems that manage them all [20].

Aviation: Customer data, as well as essential airline systems, will be secured utilizing multilayer defenses to defend the infrastructure from cyberattacks. The first of these defensive layers employs preventive methods to protect airline systems from unwanted access. The detection strategy and surveillance or monitoring systems are the second defense layer, and they are used to identify security threats and analyze weaknesses in aviation systems. The third category includes active-threat response methods and countermeasures [53]. To maintain the security of these systems and the information they contain, authentication procedures are required [117].

Electronic commerce: E-commerce information security policy is a critical component for ensuring effective and secure service delivery [102]. Information security is achievable through secure applications of technology and other information systems for e-commerce against hostile Internet activities, and this is one effective factor toward the online business’s success. Villa et al. [28], says that information security in e-commerce should also assure the integrity, confidentiality, and availability of all such data. With the aid of authentication techniques or information encryption, it is critical to securely store and access information in e-commerce only by authorized users. To create confidence with clients, e-commerce information should also be preserved after services have been provided [131].

#### 3.2.8. Access Control Policy

In financial institutions, educational institutions, and e-commerce businesses, access control policies are critical. In cyber settings, access control policies should be utilized to manage allowed access to accounts and prevent any unauthorized access. Through authentication techniques, access control safeguards and protects data in diverse scientific communities, academic Clouds, and university networks for educational institutions [132]. In the aviation industry, access control should be implemented to prevent abuse of surveillance systems, broadcast data to ensure privacy for location users, and data from aeronautical telecommunication networks to prevent aircraft systems from interfering with each other’s communications. The access control policy at Lowa Bank is one of the security rules in place to protect customers’ accounts from unauthorized usage. For example, rather than using an easy password, choose a tough password. In addition, access control rules were created in this company, which used ID and passwords to secure all of the company’s data. Table 9 summarizes the role of access control policy among the five enterprises.

Healthcare: To preserve patient privacy, access control measures are employed to prevent unwanted and unexpected parties from accessing and using sensitive datasets [15]. It is also used to regulate general access to healthcare providers’ health databases and information utilized for research [110]. To avoid the exposure of individual identities and protect patient privacy, semantic security and anonymity are employed for access control. One of the security concepts of the Health Insurance Portability and Accountability Act is access control policy [133].

Finance: Financial firms should prioritize access control policies that use difficult-to-guess passwords to safeguard clients’ private information and accounts from illegal access, and instead offer secure access to facilities and systems inside the sector [37]. To prevent unexpected parties from gaining access to financial firms Cloud systems, access control should be implemented utilizing semantic identification, such as fingerprint recognition [134]. It is also important to secure communication [135]. Authentication techniques typically involve inquiries about particular personal information, or even fingerprints in some situations [42].

Education: Another major problem is ensuring the confidentiality of students’ access to academic accounts and other sensitive information, as well as granting rights to only authorized users to use particular online applications at educational institutions. Due to effective authentication and authorization procedures, unauthorized users are prevented from accessing university resources. Luminita [136] states that these regulations allow administrators, teachers, and students to access educational systems based on their unique responsibilities and jobs. Furthermore, access control rules are used to monitor students who utilize university networks to obtain information and knowledge [22]. Authentication methods and passwords can aid in the management of remote access to the library and online applications by students, employees, and professors [37].

Aviation: In the aviation industry, access control policy is critical for regulating access by people, vehicles, luggage, and cargo, as well as securing connected technological systems [58]. The other critical issue in aviation infrastructure is ensuring the security of critical areas within airport vicinities, by relying on access control policies and other biometric technology to recognize unauthorized individuals among airport workers to ensure the security of critical areas within airport vicinities [25]. In addition, access control techniques can aid in the protection of passenger data stored on aviation Cloud servers from unauthorized persons [25].

Electronic commerce: The security of e-commerce is ensured by the authentication of customers’ entries and the correct storage of relevant information, as defined by the access control policy. Strong passwords require consumers to make suitable and difficult-to-guess selections, and they must include a minimum of characters as well as the usage of symbols or digits when establishing their passwords. To provide safe e-commerce services and online websites, complex login is employed [102]. Furthermore, access control rules are helpful tools for dividing access control into different levels based on the tasks that each group is responsible for; for example, customer affairs management should not have access to payment affairs [101]. These policies can also be used to restrict consumer access to card payment information and limit it to authorized persons. Access control should also be used for online computing resources and wireless e-commerce infrastructures, as well as physical resources such as servers, storage, and switches [37].

#### 3.2.9. Data-Retention Policy

To create trust and confidence with consumers, every business dealing with and maintaining large volumes of financial reporting should comply with and embrace the Sarbanes–Oxley Act. This statute also forbids modifications to corporate data that are retained, ensuring data integrity. To preserve privacy, healthcare institutions should establish a data-retention policy and store personal health information for set periods. A retention plan should be established in the aviation sector to keep passenger name information anonymous and prevent it from being deleted. Furthermore, to safeguard the privacy of alumni data and records, all institutions should comply with data retention rules. Wesleyan University is one of the institutions that have data-retention policies in place, such as not keeping any student data. Additionally, after 120 days, it inhibits the retrieval of deleted data from shared network devices and file locations, as well as the retention of user data that has been erased from the user system and student’s email [137]. Table 10 summarizes the role of the data retention policy among the five enterprises.

Healthcare: There are regulations in place for data retention for specific periods, and personal health information must be saved [138]. Furthermore, when medical treatments are completed, every health organization should discard and dispose of health data [15]. To minimize inadvertent exposure, any identifying information about individuals, as well as health directives such as personal information about patients or medical personnel, test results, hospital operations, and medical treatment fees, should be removed from hospital databases [137].

Finance: Financial institutions’ needs for the preservation of yearly reports, payment records, and account payables and receivables, as well as the retention of personal information for consumers, should be emphasized in retention policies [47]. Customers’ credit card information, as well as other sensitive data, should be maintained only for a limited time, and encrypted while stored to avoid inadvertent exposure. If there is no longer a need for data, they should be deleted [137].

Education: For university students, it is critical to implement data-retention regulations. If there is no need to keep the alumni data, they should be deleted as soon as possible. Students’ permanent records must be preserved for as long as the student is enrolled in classes [84]. The student information that is kept on file should be accurate and up to date. Alumni data and records must be discussed with them in order to confirm the status of their data retention [139]. To maintain privacy and security for students, identifiers or sensitive content should be removed from university web addresses [137].

Aviation: Traveler information, passenger names, and other records should be kept for a certain amount of time [140]. In addition, retention regulations should aid to prohibit the retention and copying of scanner pictures by anybody at airports, to protect passengers’ privacy [127]. Furthermore, passenger name records and other profiles can be retained for just a limited time, masking all aspects that can be identified in each traveler [141]. Furthermore, passenger data do not need to be deleted in the traditional sense but rather anonymized [142]. Finally, only authorized individuals should have access to any preserved passenger data [112].

Electronic commerce: Customers’ data-retention rules prohibit the storage of credit card numbers and expiration dates, as well as limiting the period for such storage [101]. In addition, all prior customer records should be removed from the database, leaving only a minimal number of customer records required for transactions [84]. Furthermore, data backup procedures should be in place if hard drives fail or viruses infiltrate computers [102]. The Fair Credit Reporting Act, which limits the retention of consumers’ credit information to certain periods, should be followed by e-commerce industries [137].

#### 3.2.10. Data-Protection Policy

Enterprises should have data-protection regulations in place to secure all personal information, which is divided into two categories: personally identifiable information and personally nonidentifiable information. Furthermore, there is evidence that all businesses should adopt the European data-protection regulatory principles to secure their consumers’ personal data [143,144]. The findings also suggest that explicit data-protection regulations should be in place to secure individuals’ personal data, as well as consequences for data breaches. The Barnsley Hospital is one of the businesses that follow the principles of the Data Protection Act of 1998 to guarantee that the personal data of patients are used fairly and properly [121]. Table 11 summarizes the role of data-protection policy among the five enterprises.

Healthcare: When dealing with patient’s personal information, there should be a high level of protection. It must, for example, be handled equitably and exclusively for defined reasons by health organizations, as well as securely kept and sent [15]. Only authorized personnel should have access to this type of information, and it should only be used for the main reasons [121]. Personal data must be managed in the health sector by arrangements between data owners to share with other entities, with penalties for non-compliance in the case of violation [145].

Finance: Customers’ personal information must be safeguarded at financial firms. For example, information such as names, contact information such as email and phone numbers, and geographical data such as addresses [146]. Financial institutions shall not utilize personal information about their clients for any reason other than the one for which it was acquired [146]. If there are incidents of improper management or access to such sensitive material, disciplinary action should be taken [45].

Education: Personal data of students, employees, and faculty must be protected and used for particular reasons in a timely way in all educational institutions and universities, and there should be disciplinary procedures in place if these security measures are breached. Mthunzi et al. [10] describe how the procedure involves secure storage and processing of everyone, whether within or outside the university’s facilities, including those stored in Cloud settings. Students’ data should be collected, handled, stored, and destroyed securely. Furthermore, secure communication and viewing data on the university’s website should be made available. Secure disclosure can be accomplished by obtaining the person’s written authorization. This protection extends not just to personal information but also research initiatives [45]. Another issue is to secure students’ personal information by employing access control methods such as passwords [62].

Aviation: Protecting passenger personal data is equally as important in the aviation industry, and it should only be used for specified objectives [25]. Furthermore, safeguards must be in place to prevent illegal access to passengers’ personal information. Such data require secure gathering, and if data protection standards are not followed in aviation systems, accountability steps must be implemented [95]. The encryption of passenger data obtained by airlines is another data protection measure [147].

Electronic commerce: Comprehensive data-protection regulations are needed in e-commerce, to govern consumer data all around the world. These regulations ensure that consumer data are handled legally, whether they are collected, disclosed, processed, or transmitted [148]. These regulations, on the other hand, prohibit the publication of consumer information in e-commerce [52]. Integrity, privacy, and security of client data are all aspects of data protection in e-commerce. Only consumers, for example, have the right to data portability and the transfer of a copy of their data from one supplier to another [86].

## 4. Discussion

This study intends to address two research questions: what are the common characteristics of CS policies utilized by different companies? (RQ1), and how are CS policies used by different firms? (RQ2). RQ1 was answered in Section 3 and RQ2 was answered in Section 4. In this section, the implications of the findings as well as the limitations of this study are discussed.

### 4.1. Implications

This paper identified 10 common CS policy aspects in five enterprises: healthcare, finance, education, aviation, and e-commerce. However, these findings also show that these enterprises differ in terms of how important CS policies are to them. The differences are due to the nature of the information controlled by these businesses as well as the business needs. Because of the sensitive nature of the information handled, privacy policy appears to be a high priority among all of these enterprises. This is consistent with Schwartz and Solove’s [149] findings, which define privacy laws as rules that protect personally identifiable information but not non-personally identifiable data. Furthermore, the findings revealed the role of CS policies to be critical in ensuring the security of Cloud computing in these enterprises.

According to the findings, website CS rules are a major problem in the banking and e-commerce industries. Security influences the quality of financial services that attract clients, according to Kaya [150], and is essential for client happiness [37]. E-commerce security, according to Hartono et al. [151], is a crucial problem for establishing consistency and strengthening customers’ confidence and expectations. Customers are also cautious to shop online owing to security concerns, according to the study, and high levels of website security was related to increased intent to buy online. Furthermore, increasing website security and quality can lead to continuous usage of e-commerce websites, and such security not only enhances consumer trust but also mitigates possible dangers in the e-commerce environment [37]. Furthermore, the findings revealed the importance of email security in all of the previously mentioned industries. This is in line with previous [129,152,153] findings. According to Kigerl [154], to guarantee the security of commercial emails, the CAN-SPAM Act should be obeyed, with the act’s enforcement function being to define the standards for commercial emails transmitted between consumers and businesses to avoid email spam [113].

According to the findings, physical security regulations are also crucial in the banking, health, and aviation industries. This is per Yusta et al. [155], who state that infrastructure can result in loss of life or significant negative impacts on health and safety, the economy, and national security if interfered with or destroyed [45]. Furthermore, all of the investigated companies’ network security practices are serious. This is per Talib and Alomary [108], who also stressed the need for adhering to the Cyber Network Act to strengthen defensive measures in these businesses. Moreover, information security is a critical issue in all studied enterprises, as stated by Peltier [17], who stated that every business must have effective information security policies in place to protect their valuable data, and that businesses should follow the ISO 17799 standard as their information security policy or ISO 27001 as an industrial and commercial standard, and that businesses should follow the ISO 17799 standard as their information security policy [60].

The findings also revealed that both the educational and financial sectors require access control policies. This is in line with the findings of Demchenko et al. [156] and Li et al. [134], who emphasized the importance of providing authentication of online transactions to protect customers’ financial information. In addition, the importance of data retention policies in the healthcare, finance, education, e-commerce, and aviation sectors is also highlighted by the findings. This is per Greene [138], who stressed the necessity of following data-retention regulations to protect e-personal health information. To protect patients’ privacy, the author further notes that electronic personal health information should be kept for only a limited time before being discarded, and that data-retention policies for health information should adhere to the Health Insurance Portability and Accountability Act. According to Hasan and Winslett [157], the Sarbanes–Oxley Act governs this procedure, to protect the integrity of company records and the retention of immutable copies of emails, spreadsheets, and financial documents. On the other hand, the findings revealed that data-protection policies are a critical issue for all businesses. This is consistent with the findings of [116], who emphasized the importance of data-protection offices in any organization, as well as the importance of all employees and customers being informed on how to protect their personal information [146].

To construct a secure infrastructure for any customer–server enterprise, some recommendations to the security branches within each enterprise can be made based on the analysis.

- It should incorporate CS policies appropriate to the industry. Each company should set up a CS office to oversee the security of their information and communication infrastructure, with primary responsibility for implementing CS policies in both technical and administrative elements of their operations, such as using IDs, firewalls, and cryptography;
- To track and oversee the execution of these security rules in each sector, all companies’ information and communication infrastructures require leadership and follow-up processes;
- The policies should be understood and approved by all workers. Each security strategy should be tailored to a specific group of people. A team should be formed within the organization to train employees on the company’s CS policies, with a focus on how to protect against spam and phishing emails;
- It should be written in accordance with the company’s culture, such as its educational, financial, and medical policies. Furthermore, CS rules should be adjusted in response to changes in the severity of threats over time;
- Network-related policies, data-protection-related policies, Cloud policies, and other policies will be used to create security policies;
- Effective, useful, consistent, relevant, useable, intelligible, legible, easy, and memorable CS policies are essential;
- Each security policy must meet a distinct requirement and must thus be maintained individually.

### 4.2. Limitations and Future Work

Although this research addresses the research questions in hand, and despite all of the efforts put into its preparation, there are some unavoidable limitations. To begin with, this study does not cover the CS of all enterprises. This study is limited to five enterprises (healthcare, finance, education, aviation, electronic commerce) which are significant for digital society and people. However, there are some others, for instance: smart grid, telecommunications, manufacturing/Industry 4.0, Logistics 4.0, etc.

Furthermore, more fieldwork or a survey of some current businesses could have improved this study’s understanding of what security requirements exist in other businesses. Accordingly, there are numerous possibilities for expanding the scope of this research in various directions. One way is to increase the number of businesses by including industries such as communication. Moreover, some fieldwork and interviews with each enterprise’s security policy office, as well as questionnaires or surveys to supplement the data for analysis, will be more successful. Another option for the future is to establish official laws or rules for each enterprise’s CS that does not presently have any. Finally, it is expected that this research will serve as a steppingstone for the establishment of more comprehensive CS policies in the future, particularly as future companies require higher levels of network security and operational processes.

Underestimating the need for cybersecurity knowledge exposes all of the company’s assets to a significant danger. Users are likely unaware of the many forms of CS policies, which have a big impact on their awareness of cybercrime [158]. As a result, workers’ activities may have an impact on the firm’s cybersecurity projects’ success or failure. All stakeholders must be aware of the CS policies, since this awareness may lead to the adoption of appropriate behaviors [30]. Moreover, to create adaptive defensive methods against many sorts of attacks, adequate attention and deep debate in the field of diverse attack techniques is required [159]. Future research may investigate how enterprises develop policies against these diverse attacks.

Few companies structure security management and incident response in such a way that they can respond to security occurrences and proactively navigate the threat setting by learning from their experiences [160]. Information security management and incident-response operations that are more integrated are better equipped to safeguard digital assets. Organizations may better adjust their security policies to the threat environment when there is a strong connection between security management and incident response. A weak connection, on the other hand, leads security defenses to stagnate, impeding the organization’s capacity to fulfill existing security goals or establish new, more suitable ones [160]. The stronger the organization’s security performance, the more learning opportunities it has. Because of this link, further research routes may be studied [30].

## 5. Conclusions

With growing digital transformation, ICT is now widely used in a wide range of business domains. However, there have been numerous issues with CS. These issues divert our attention towards an increased need for safeguarding organizations’ ICT infrastructure. CS is an important factor to consider if organizations want to keep their customers’ information safe from cybercriminals and malicious programs on the Internet. The objective of CS is to guarantee that systems are secure, reliable, and available. Security policies that protect an enterprise’s cyberspace are one example of such security measures. The current study investigated and discussed the various customer service policies in place to ensure that the customer’s information is effectively managed and that their expectations are met. We have also looked at common customer service policies from various businesses’ viewpoints; each of these companies has a unique approach to customer service. The five industries that were discussed were health, finance, education, aviation, and e-commerce. We aimed to build a strong infrastructure in each business and to look into the security laws and policies that apply to all businesses in each sector.

Privacy, website security, Cloud computing security, email security, physical security, network security, information security, access control, data retention, and data protection, were all found to be common among the five kinds of businesses enterprises, with the privacy policy being the most key element to protect sensitive information. Some CS policies were determined to be more essential when compared to others. The Family Educational Rights and Privacy Act, which protects personal student information, the Gramm–Leach–Bliley Act, which protects financial information, the Fair Credit Reporting Act, which protects credit information, and the Health Insurance Portability and Accountability Act, which protects personal health information, have all been identified as critical to CS. In addition, a balance between human rights protection and security measures, such as preserving any personal information exposed as a result of the deployment of body-scanner equipment at airports, should be found. Furthermore, all of their client’s personal information, which comes in two forms: identifiable information and nonidentified information, should be protected by data-protection regulations.

## Figures and Tables

**Table 1:** Comparison matrix of common CS aspects for different enterprises (Red = Very High (VH), Green = High (H), Blue = Average (AVG), Yellow = Low (L)).

Common Attribute	Healthcare	Finance	Education	Aviation	E-Commerce
Privacy policy					
Website policy					
Cloud computing policy					
Email policy					
Physical policy					
Network policy					
Information policy					
Access control policy					
Retention policy					
Data protection policy					

**Table 2:** Privacy policy among different enterprises.

Sector	Privacy Policy
Healthcare [15,71,88,89]	Guidelines to secure patient information from unauthorized collection, disclosure, processing, or transmission. Comply with HIPAA Act.
Finance [71,90,91]	Guidelines to protect customer’s information and use or process them in legal ways. Prevent financial information from being disclosed without the approval or knowledge of clients. Comply with GLB Act.
Education [47,92,93]	Guidelines to protect sensitive data of the student such as health information, grades, disabilities, psychiatric problems, and personal and academic conduct from being disclosed without the students’ agreement. Comply with FERPA laws.
Aviation [25,94,95,96]	Guidelines to protect passenger’s data that show in body-scanner devices in airports from disclosure without his/her knowledge.
E-commerce [28,86,88,97,98]	Customers’ financial information, such as credit card information, is protected from disclosure, use, and processing without their consent or knowledge. Comply with Fair Credit Reporting Act.

**Table 3:** Website security policy among different enterprises.

Sector	Website Security Policy
Healthcare [4,15]	Guidelines to prevent any cross-site scripting and clickjacking.
Finance [42,99]	Define secure web applications and software to design websites of financial institutions to prevent code-injection attacks.
Education [47,74]	Define secure web applications and software of academic websites and faculty and student sites to prevent code injection.
Aviation [74,100]	Define the security of web applications and software of airline websites to prevent code-injection attacks and malicious content.
E-commerce [73,101]	Define the security of web applications and software of e-commerce websites to protect against code injection.

**Table 4:** Cloud computing security policy among different enterprises.

Sector	Cloud Computing Security Policy
Healthcare [1,103]	Authentication methods, firewall devices, and other security measures are used to safeguard Cloud computing equipment to allow a secure connection between patients and servers providers in the health Cloud.
Finance [1,104,105]	Financial firm Cloud protection guidelines to offer secure services between clients and financial organizations, utilizing all available security measures to safeguard data, applications, and transactions.
Education [10,83]	Guidelines to safeguard data, applications, transactions, data server providers, academic programs, and secure connections between students and universities or educational institutions.
Aviation [106,107]	Using all security measures to safeguard data, applications, transactions, reservation applications, and offer secure connections between airports and clients.
E-commerce [102,108]	Authentication methods, firewalls, IDs, and intrusion-prevention systems are used to safeguard data, services, and applications to provide secure commercial services and secure financial transactions. Using ID as an electronic identity and signature service over the Internet.

**Table 5:** Email security policy among different enterprises.

Sector	Email Security Policy
Healthcare [110,111]	Guidelines to ensure safe contact between patients and medical institutions by advising on how to use emails properly, such as avoiding dangerous attachments and utilizing antispam software, or avoiding opening executable programs.
Finance [101,104,112]	When consumers and financial businesses communicate through email, guidelines to offer secure communication techniques through the correct usage of emails and digital signatures are provided.
Education [79,113]	Guidelines for utilizing emails properly to ensure safe contact between students and institutions, such as avoiding phishing messages, questionable links, and attachments from anonymous addresses, and employing spam filters.
Aviation [25,113,114]	Guidelines for providing safe communication for passengers and airline reservation services, as well as the use of software filters to prevent spam emails and dangerous files.
E-commerce [102,115]	Guidelines for providing safe communication for passengers and airline reservation services, as well as the use of software filters to prevent spam emails and dangerous files.

**Table 6:** Physical security policy among different enterprises.

Sector	Physical Security Policy
Healthcare [59,116]	Guidelines to protect all physical assets of health sectors such as buildings of hospitals, medical devices, and network equipment by using physical and digital locks.
Finance [17,80]	Guidelines to protect physical assets, ATMs, physical buildings, or saving rooms from being destroyed or invaded by using physical and digital locks.
Education [80,92]	Guidelines to protect academic buildings and universities’ lab equipment and classrooms, learning and teaching materials of educational institutions by using physical and digital locks.
Aviation [5,25,117]	Guidelines to protect physical assets of the aviation sector such as airport traffic control towers and surveillance devices by using physical and digital locks.
E-commerce [52,98,115]	Guidelines to protect IT resources of e-commerce and online business by using physical and digital locks.

**Table 7:** Network security policy among different enterprises.

Sector	Network Security Policy
Healthcare [120,121]	Using several security layers such as firewalls, IDS, and IPS, guidelines to safeguard computer and network devices, traffic networks, and enable secure contact between patients’ end-points and physicians or nurses.
Finance [74,122,123]	Guidelines for securing computers and network devices in financial organizations by employing several security layers to enable secure transmission between two PCs.
Education [15,124]	Multiple security layers are used to safeguard computers and network devices at educational institutions, allowing for secure connections between PCs.
Aviation [37,125,126]	Guidelines to protect computers and network devices to provide secure communication between PCs in the aviation industry by many levels of security.
E-commerce [102,120]	Multi-layer security guidelines to safeguard computer network devices and allow a secure connection between PCs in commercial services.

**Table 8:** Information security policy among different enterprises.

Sector	Information Security Policy
Healthcare [15,128]	Guidelines to safeguard all sorts of data, including medical knowledge, statistics, and research data, as well as patient visits.
Finance [4,129,130]	Banks and financial organizations should follow these guidelines to secure all of their information and information systems.
Education [20,22]	Guidelines to safeguard information handled by universities and IT systems, as well as all other types of information such as academic data and scientific research, as well as the systems that process these data.
Aviation [53,117]	Guidelines for utilizing detecting devices and monitors to secure all airports’ IT and aviation systems.
E-commerce [28,102,131]	To earn consumer trust, guidelines to protect all uses of technology and information systems are needed to deliver secure commercial services.

**Table 9:** Access control policy among different enterprises.

Sector	Access Control Policy
Healthcare [15,110,133]	To protect illegal access to patient data and health systems, as well as medical applications, mechanisms are in place. Comply with HIPAA regulations.
Finance [37,42,134,135]	Mechanisms to create strong authentication to control access to financial services to protect accounts and confidentiality of customers’ data.
Education [22,37,136]	Mechanisms to manage student or faculty access to university educational websites, regulate student access to their academic sites, and prevent illegal entry.
Aviation [25,58]	Mechanisms for preventing illegal access to aviation services or airport areas by managing access control and providing authentication.
E-commerce [37,101,102]	Access control mechanisms, such as strong passwords, are used to access business websites and conduct financial transactions.

**Table 10:** Data-retention policy among different enterprises.

Sector	Data-Retention Policy
Healthcare [15,137,138]	Rules to govern the secure preservation and destruction of patient data once medical treatments are completed; specify the time limits for which personal data of patients may be saved.
Finance [47,137]	Rules to ensure secure preservation of customer data, yearly reports, and payment records by encrypting data and deleting client data for old clients when they are no longer needed. Comply with Sarbanes–Oxley Act.
Education [84,137,139]	Rules for retaining information on students and alumni in a timely and secure manner, as well as the secure disposal of data relating to former pupils in order to ensure long-term security.
Aviation [112,127,140,141,142]	Rules for the secure preservation of passenger name records by encryption and anonymized archiving of any old passenger data, as well as for avoiding the storage of passenger scanner pictures.
E-commerce [84,101,102,137]	Rules to ensure secure retention of clients’ data, restrict the keeping of financial information such as credit card numbers and credit expiration dates, or only store them for certain durations, and ensure a safe backup of clients’ data are in place. Comply with Sarbanes–Oxley Act.

**Table 11:** Data-protection policy among different enterprises.

Sector	Data Protection Policy
Healthcare [15,121,145]	Patients’ data, including identifiable and nonidentified information, are handled and collected according to a set of rules.
Finance [45,146]	Customers’ data, such as names, contact information, such as email and phone numbers, and geographical information, such as addresses, are processed and handled according to certain rules.
Education [10,45,62]	Rules to protect students, staff, and faculty data, as well as secure handling and access to students’ data.
Aviation [95,147]	Passengers’ data are protected by rules that govern the secure processing of identifiable and nonidentified information, such as passport numbers, names of passengers, travel destinations, and so on.
E-commerce [52,86,148]	Personal data about consumers acquired from commercial sites and online business services are handled transparently and protected.

## Appendix A

**Table A1:** Surveyed papers significance and focus.

Reference	Significance	Security Policy
[70]	An overview of the issues and necessity for critical infrastructure protection, as well as the worldwide hazards that are associated with it	Privacy policy
[71]	Outline the current situation of privacy law in the USA and how it differs from other nations in terms of substantive safeguards	
[72]	Examining how the legislative systems in Europe and the USA aid in the protection of sensitive customer data on the Cloud	
[88]	Privacy and data-protection regulations	
[89]	End-to-end privacy and security control for collaborative access to healthcare	
[90]	Coordination between IT, business, and information security initiatives is addressed by an integrated framework	
[91]	The European Union (EU) General Data Privacy Regulation (GDPR) to address difficulties linked to personal data protection and to unify data protection across the EU	
[92]	Family Educational Rights and Private Act (FERPA) in the USA to protect students’ personal privacy while also making their educational data available to them	
[93]	Balance student’s privacy, anonymity, and big data in the social sciences	
[94]	Personalization of web pages for online users	
[96]	Law enforcement and trusted traveler programs are blurring the lines between privacy and security	
[38]	A conceptual model that blends the Unified Theory of Acceptance and Use of Technology (UTAUT) with risk perception to analyze online banking behavior intention and utilization	Website policy
[73]	Multidomain research of phishing-website detection technologies to calibrate the trust of automated security IT artifacts	
[75]	A model to enhance social media access control systems	
[99]	Explore the methods of security used by websites—how should we safeguard our data, and what is the best course of action?	
[100]	Delivers up-to-date research on customer sentiments, beliefs, thoughts, intentions, and attitudes toward effective customer interaction management	
[1]	A theoretical framework for cybersecurity management in Cloud computing based on a semantic review of the literature	Cloud policy
[76]	A model for dealing with the issues of data-intensive applications on mobile Cloud platforms	
[77]	Addresses data security problems and solutions in Cloud computing paradigms including delivery of services and deployment strategies	
[83]	A study model that merged aspects of information systems security policy	
[105]	Discuss why banks need to implement additional security measures in addition to the protection supplied by the cloud vendors	
[106]	Discussion of the use of cloud computing in different aviation and aerospace businesses, as well as the growing technology concerns	
[107]	Using a digital signature with the RSA encryption method to improve the data security of cloud computing	
[108]	A model to integrate Cloud computing and e-commerce as a service	
[103]	Examine the privacy and security concerns created by the usage of the Cloud in e-health	
[46]	Invention applications and the USA patent and trademark office’s success	Email policy
[78]	An overview of the law at USA schools and security challenges	
[79]	Using email user insights to guide organizational email management policies	
[110]	Evaluation of employee sensitivity to phishing attacks in US healthcare facilities	
[111]	Discussion of the best standard for email encryption for Health Information Technology for Economic and Clinical Health (HITECH) Act compliance to secure Protected Health Information (PHI)	
[112]	Study on the role of Russian “patriotic hackers” in the cyberattacks on Estonia and Georgia in 2007 and 2008, and how the expertise of surviving this attack aided	
[113]	A new approach for detecting fake customer reviews	
[114]	A machine-learning-based approach for detecting spam with high accuracy	
[5]	Comparison between the United Kingdom and France in terms of safeguarding key infrastructure from cyberattacks	Physical policy
[12]	An explanation of the distinctions between information security and cybersecurity	
[17]	Guidelines for information security standards, procedures, and policies	
[59]	Examining information security policies and objectives and offering insights on them through a rigorous theoretical perspective	
[80]	Explanation of physical security and information security, and why treating these two distinct forms of security in a unified manner is vital in today’s shifting security scenario	
[98]	A theoretical model that studies the link between security, perceived risks, and privacy, and how they relate to customers’ confidence in e-commerce	
[116]	Providing baseline information on the Data Protection Officer (DPO) in order to ensure data security and compliance	
[24]	A survey on privacy protection and data security for Cloud storage	Network policy
[51]	An overview of cybersecurity and networks that emphasizes their linkages and the complexity of current network design challenges	
[81]	An examination of the smart grid’s IoT-based security issues and potential remedies	
[120]	A review of security vulnerabilities in wireless medical sensor network-enabled healthcare applications	
[121]	Review of the Directive 2011/24/EU on patients’ rights in cross-border healthcare	
[122]	Discussion of the concept and development of The Interface to Network Security Functions (I2NSF) architecture, providing ways to improve its efficiency through integration with SDN	
[123]	Network security concepts and guidelines	
[124]	Review of risks and remedies in e-learning systems	
[125]	Outlines the primary variables that underlie the United States’ attempts to improve their cybercapacity	
[126]	Exploring an innovative approach to privacy protection and providing a persuasive collection of prospective legislative suggestions and practical answers	
[20]	A comprehensive framework that covers all areas of online learning design, creation, and planning	Information policy
[28]	A bibliometric investigation of the structure, quality, and quantity of e-commerce usage in emerging economies’ small- and medium-sized businesses	
[53]	Analysis of security flaws in the Automatic Dependent Surveillance-Broadcast (ADS-B) system and suggestions to improve security	
[57]	An overview of current and future challenges facing behavioral information security	
[82]	An overview of information security policy violations by employees in corporate contexts	
[128]	A model, using a Chaotic Map-Based Three-Factor Authenticated Key Agreement Scheme to Secure Telecare Medical Information Systems	
[129]	An overview of the electronic banking service, stressing several security concerns and proposing potential solutions	
[130]	An overview of remote working software professionals’ perceptions on the Information Security Policy (ISP) and the factors that should be considered in order to keep remote employees in the software sector productive	
[131]	Discussion about Saudi Arabia’s e-government vulnerabilities and the necessity for a defined information security system	
[43]	Evaluation of the Cloud security by defining specific security needs and solutions that overcome possible threats	Access control policy
[58]	A framework that enables academics to improve their efforts in the field of insider attacks	
[133]	A model to enhance privacy and access control in electronic health-record systems	
[134]	A strategy for obtaining safe banking services on multimedia large data in Cloud computing	
[135]	An overview of online banking communication and authentication security issues	
[136]	Elucidate the main security concerns that must be addressed while creating while using an e-learning system	
[47]	An overview of essential challenges of big data, such as technological difficulties, a lack of expertise, and epistemological and ontological disparities	Data-retention policy
[84]	An overview of privacy rights and regulation of personal data retention and erasure in European Union (EU)	
[85]	A book of the illegal and legal considerations of information security	
[137]	Two models for standardizing audit query meaning	
[138]	A model of HIPAA compliance regulations for clinician texting	
[139]	A policy framework to create an environment that allows for ethical data collection and use, and to address concerns of susceptibility	
[140]	An overview of European security laws in European passenger name recording system	
[141]	Discussion of the European proposal to share the personal information of all passengers	
[142]	An assessment of the transnational problems faced by government monitoring and business interference on the right to privacy	
[62]	A book on information technology crimes and regulations	Data-protection policy
[67]	An overview of the privacy problems of wearable personal health-monitoring equipment	
[86]	A book on e-commerce, IT, and data protection in Europe	
[145]	E-health, privacy, IoT, design, and corporate compliance framework	
[147]	An examination of the primary data-sharing tools utilized by police agencies and intelligence agencies in the EU and the US was conducted between 2001 to 2015	
[148]	A review of the privacy of personal data in the Malaysian setting	
[4]	A review of the evolution of information security policy	Website and information policies
[10]	Evaluation of Cloud security concerns and designing preventions or remedies, depending on the source or cause of a security issue	Cloud and data-protection policies
[15]	A book on the development of information security in healthcare	Privacy, network, information, access control, data protection, and data-protection policies
[22]	A review of cybersecurity metrics and their applications in e-learning systems	Information and access control policies
[25]	An investigation into the ethical concerns surrounding the aviation industry in Indonesia	Physical and access control policies
[37]	Evaluation of the access controls applied on IoT, including the adoption of access controls and the challenges of access control techniques	Network and access control policies
[42]	An assessment of Cloud computing security and privacy, as well as a proposal for a framework of possible solutions	
[45]	An overview of the European Union’s General Data Protection Regulation (GDPR)	Privacy, website, and data-protection policies
[74]	Evaluation of security design methods and their qualities, we used a hybrid technique called Fuzzy AHP-TOPSIS (Analytic Hierarchy Process-Technique for Order Preference by Similarity Ideal Solution)	Website and network policies
[95]	Examining the background and future possibilities of body scanners vs. data protection and privacy via the lenses of challenges, legal tools, and potential remedies	Privacy and data-protection policies
[158]	A review of how small- and medium-sized businesses manage cybersecurity risks	Website, email, access control, data-retention policies
[102]	A review of e-commerce security challenges and solutions	Cloud, network, information, access control, physical, and data-retention policies
[104]	A book of CompTIA Advanced Security Practitioner (CASP) certificate guidelines	Cloud and email policies
[115]	Analysis of e-commerce and mobile commerce (M-Commerce) security issues and solutions	Email and physical policies
[117]	A book on public aviation law that covers international safety requirements, safety rules, and security regulations, among other aviation topics	Physical and information policies
